# Defective excitation-contraction coupling is partially responsible for impaired contractility in hindlimb muscles of *Stac3* knockout mice

**DOI:** 10.1038/srep26194

**Published:** 2016-05-17

**Authors:** Xiaofei Cong, Jonathan Doering, Robert W. Grange, Honglin Jiang

**Affiliations:** 1 Department of Animal and Poultry Sciences, Virginia Tech, Blacksburg, VA, USA; 2 Department of Human Nutrition, Foods, and Exercise, Virginia Tech, Blacksburg, VA, USA

## Abstract

The *Stac3* gene is exclusively expressed in skeletal muscle, and *Stac3* knockout is perinatal lethal in mice. Previous data from *Stac3*-deleted diaphragms indicated that *Stac3*-deleted skeletal muscle could not contract because of defective excitation-contraction (EC) coupling. In this study, we determined the contractility of *Stac3*-deleted hindlimb muscle. In response to frequent electrostimulation, *Stac3*-deleted hindlimb muscle contracted but the maximal tension generated was only 20% of that in control (wild type or heterozygous) muscle (*P* < 0.05). In response to high [K^+^], caffeine, and 4-chloro-m-cresol (4-CMC), the maximal tensions generated in *Stac3*-deleted muscle were 29% (*P* < 0.05), 58% (*P* = 0.08), and 55% (*P* < 0.05) of those in control muscle, respectively. In response to 4-CMC or caffeine, over 90% of myotubes formed from control myoblasts contracted, but only 60% of myotubes formed from *Stac3*-deleted myoblasts contracted (*P* = 0.05). However, in response to 4-CMC or caffeine, similar increases in intracellular calcium concentration were observed in *Stac3*-deleted and control myotubes. Gene expression and histological analyses revealed that *Stac3*-deleted hindlimb muscle contained more slow type-like fibers than control muscle. These data together confirm a critical role of STAC3 in EC coupling but also suggest that STAC3 may have additional functions in skeletal muscle, at least in the hindlimb muscle.

Skeletal muscle contraction is essential for movement, posture, heat generation, and respiration in mammals. Skeletal muscle contraction begins with the transmission of action potentials from the motor nerve causing the release of acetylcholine (ACh) from the pre-synaptic terminals. The released ACh binds to its receptor (AChR) on the sarcolemma^1^,^2^. Sodium influx through the AChR depolarizes the sarcolemma and subsequently causes the generation of action potentials. Action potentials spread along the transverse tubules (T-tubules) into the muscle fibers^3^,^4^. It is believed that in response to action potentials transmitted from the T-tubules, the dihydropyridine receptors (DHPR) on the T-tubules change their conformation and cause the calcium channel ryanodine receptors (RyR) on the sarcoplasmic reticulum (SR) to open and release calcium into the sarcoplasm^5^,^6^,^7^,^8^. The increased sarcoplasmic calcium triggers the myofibrils to contract, completing the process termed excitation-contraction (EC) coupling^9^.

The *Stac3* gene, which encodes a protein containing a Src homology 3 (SH3) domain and a cysteine rich (C1) domain, is a member of the *Stac* gene family^10^. The *Stac3* gene is predominantly expressed in skeletal muscle^11^,^12^. Transgenic animal studies demonstrate that STAC3 is essential for skeletal muscle contraction and postnatal life in mice^11^,^12^ and that STAC3 plays an important role in skeletal muscle contraction in zebrafish^13^. A recent study demonstrates that STAC3 can target the DHPR α1 subunit to cell membrane^14^. In humans, two congenital myopathies, Native American myopathy^13^ and King-Denborough syndrome^15^, have been associated with mutations in the *Stac3* gene. *In vitro* studies using primary mouse myoblasts, C2C12 cells, and bovine satellite cells indicated that STAC3 might also play a role in myoblast differentiation^16^,^17^,^18^.

The diaphragm muscle from *Stac3* knockout mice did not contract in response to electric stimulation but generated normal tension when stimulated by the RyR agonist 4-CMC^11^. Based on this data, it was concluded that *Stac3* knockout impaired skeletal muscle contraction by disrupting EC coupling^11^. The diaphragm muscle, however, is different from limb muscles. For example, the diaphragm differs from the hindlimb muscles in the type of RyR expressed^19^. Two RyR isoforms, RyR1 and RyR3, are expressed in skeletal muscle, and they have different sensitivities to the RyR agonists, 4-chloro-m-cresol (4-CMC) and caffeine^20^,^21^,^22^,^23^. While RyR1 is the predominant RyR expressed in the hindlimb muscles, both RyR1 and RyR3 are expressed at high levels in the diaphragm^24^. The diaphragm and hindlimb muscles also differ in the type of myosin heavy chain (MHC) protein expressed. The gene encoding the major fast MHC protein *Myh4* is not expressed in the diaphragm, but it is abundantly expressed in the hindlimb muscles^25^. These and other differences between the diaphragm and hindlimb muscles prompted us to examine the effect of *Stac3* knockout on the contractility of hindlimb skeletal muscles in mice. Our data indicate that while EC coupling is impaired, it is not the sole cause for the markedly reduced contractility in *Stac3*-deleted hindlimb muscles.

## Results

### Response of *Stac3*-deleted hindlimb muscles to electrostimulation

**Figure 1:**
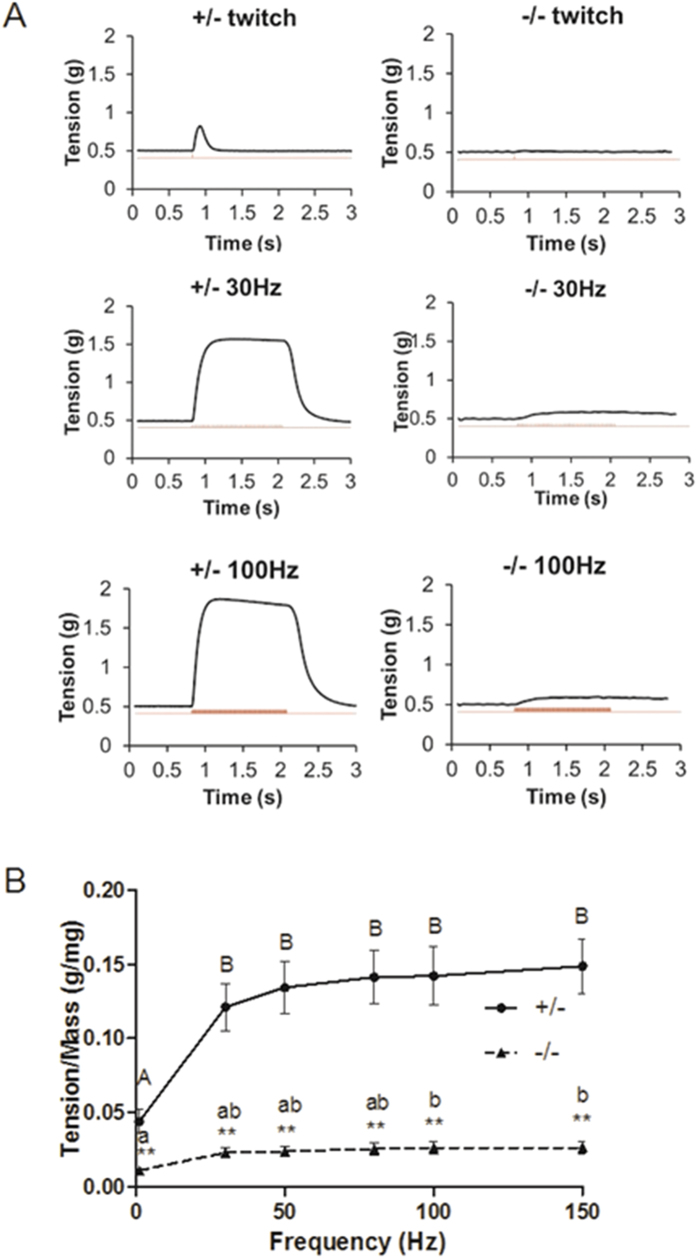
Contractile response of hindlimb muscles from E18.5 *Stac3*^+/−^ and *Stac3*^−/−^ mice to electrostimulation. Hindlimb muscle preparations were stimulated by a single electric pulse or repeated pulses of 500 μsec and 20 v, and tension was recorded with a force transducer. (**A**) Representative recordings of twitch and tetanic tension. Upper trace indicates tension changes and lower trace indicates electric stimulation. (**B**) Tension-frequency relationships. Maximum tension was normalized to muscle mass. Data are presented as mean ± SEM (n = 9 mice). **P < 0.01, between the genotypes at the same frequency. Tensions labeled with different letters are different (*P* < 0.05) within the same genotype.

When stimulated by single electric pulses, E18.5 *Stac3*^−/−^ hindlimb muscles did not contract whereas E18.5 *Stac3*^+/−^ hindlimb muscles generated twitches (Fig. 1A). When stimulated by repeated electric pulses, *Stac3*^+/−^ muscles demonstrated typical tetanic contraction (Fig. 1A). When stimulated by repeated electric pulses, *Stac3*^−/−^ muscles contracted, but the maximal tension generated was only 20% of that in *Stac3*^+/−^ muscles (Fig. 1A,B). One second after the stimulation was stopped, *Stac3*^+/−^ muscles completely relaxed but *Stac3*^−/−^ muscles remained in contraction (Fig. 1A).

### Response of *Stac3*-deleted hindlimb muscles to RyR agonists

**Figure 2:**
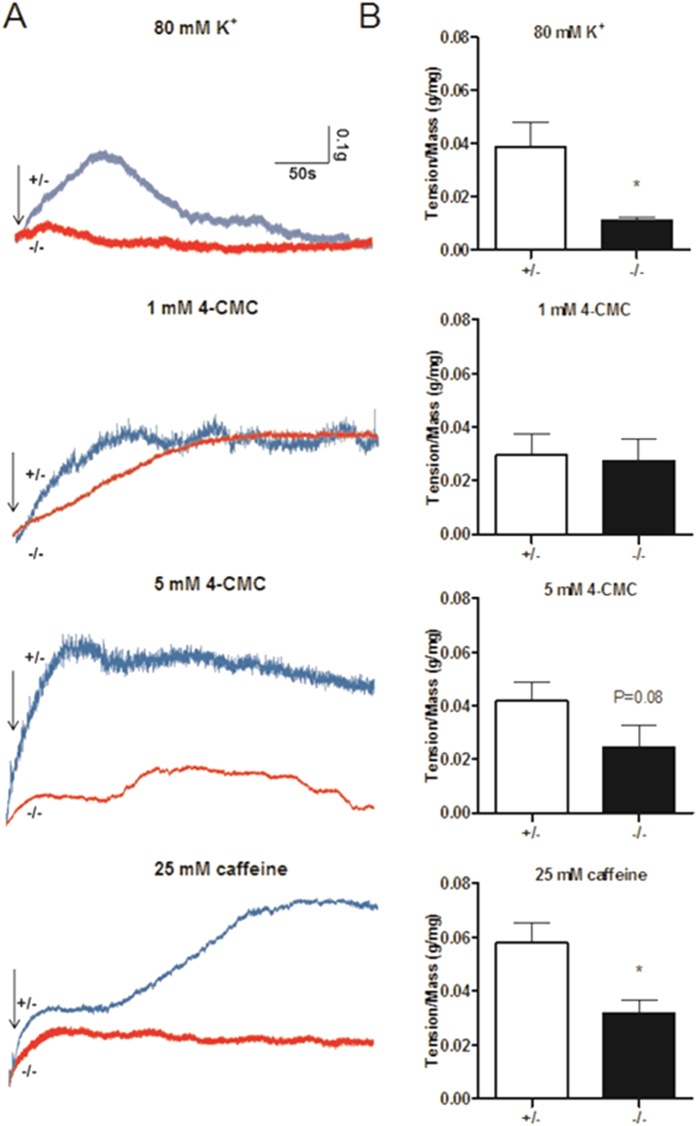
Contractile response of himdlimb muscles from E18.5 *Stac3*^+/−^ and *Stac3*^−/−^ fetuses to high [K^+^], 4-CMC, and caffeine. (**A**) Representative recordings of muscle tension. Arrows indicate when potassium (80 mM), 4-CMC (1 or 5 mM), or caffeine (25 mM) was applied to the muscle. (**B**) Maximum tension normalized to muscle mass. Data = mean ± SEM (n = 4 mice per genotype for potassium and 4-CMC; n = 5 for caffeine). *P < 0.05.

To determine the possibility that *Stac3*-deleted hindlimb muscles had defective EC coupling, we tested the contractile responses of the muscles to high [K^+^]-induced depolarization and 4-CMC and caffeine activation of RyR. Consistent with the responses to electrostimulation (Fig. 1), 80 mM [K^+^]-induced maximal tension in *Stac3*^−/−^ hindlimb muscles was only 29% of that in *Stac3*^+/−^ muscles (Fig. 2A,B). In response to 5 mM 4-CMC and 25 mM caffeine (Fig. 2A,B), the maximal tensions generated in *Stac3*^−/−^ muscles were 58% (*P* = 0.08) and 55% (*P* < 0.05) of those generated in *Stac3*^+/−^ muscles, respectively (Fig. 2A,B). These percentages were, however, much greater than those associated with electrostimulation (20%, Fig. 1) and high [K^+^] (29%, Fig. 2). Based on these percentages, nearly 60% of the lost contractility in *Stac3*^−/−^ hindlimb muscles was due to defective EC coupling (from sarcolemma to SR) while the remaining 40% was due to post EC coupling defects. The maximal tension induced by 1 mM 4-CMC in *Stac3*^−/−^ muscles was not different from that in *Stac3*^+/−^ muscles (Fig. 2). The tension induced by 1 mM 4-CMC in *Stac3*^+/−^ muscles was only 70% (*P* < 0.05) of that induced by 5 mM 4-CMC or 50% (*P* < 0.05) of that induced by 25 mM caffeine in the same *Stac3*^+/−^ muscles (Fig. 2B).

### Spontaneous and RyR agonists-induced contraction in *Stac3*-deleted myotubes

**Figure 3:**
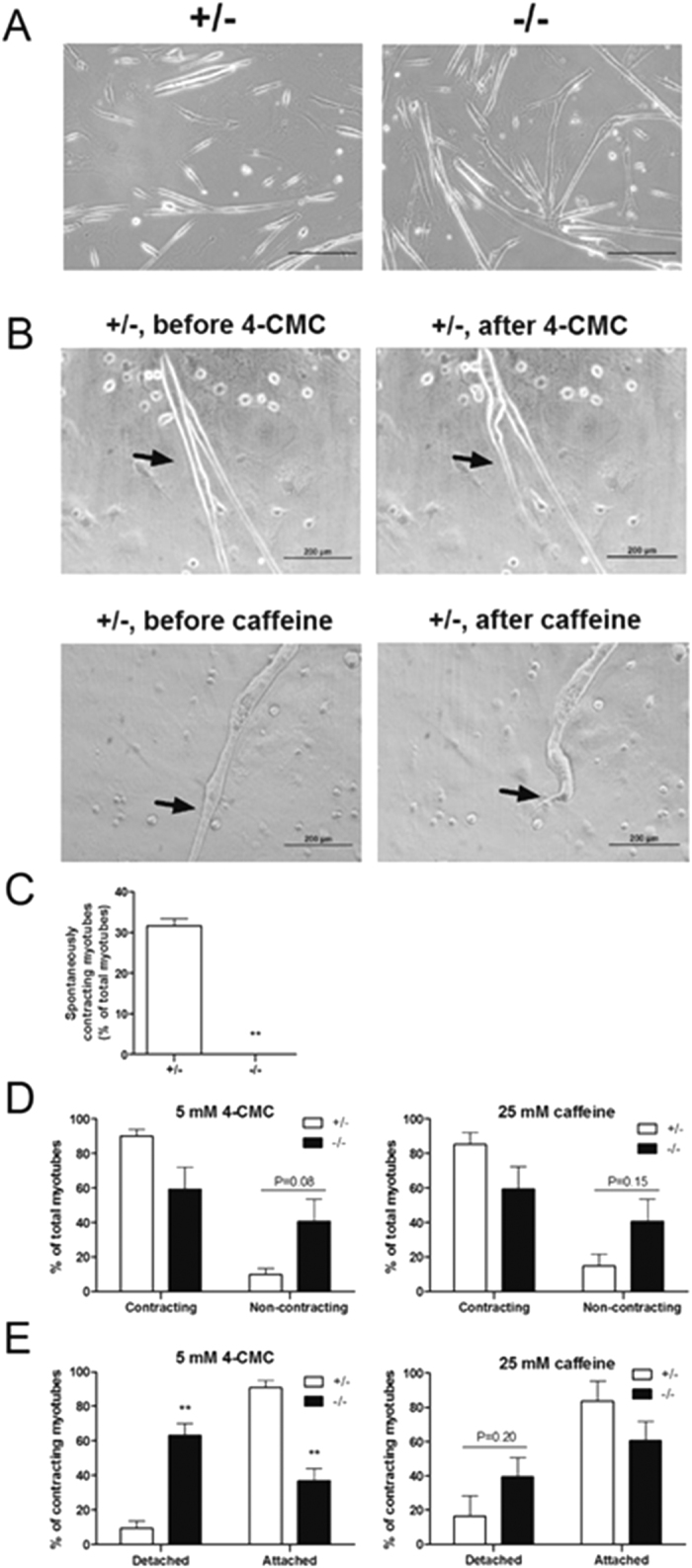
Spontaneous and 4-CMC- and caffeine-stimulated contraction in *Stac3*^+/−^ and *Stac3*^−/−^ myotubes. Myoblasts were isolated from hindlimbs of E18.5 *Stac3*^+/−^ or *Stac3*^−/−^ fetuses and induced to differentiate into myotubes. Spontaneous contraction was determined by examining 1-min videos of unstimulated myotubes. 4-CMC or caffeine-stimulated contraction in myotubes was determined by examining videos of myotubes 5 seconds before and 1 minute after the addition of 5 mM 4-CMC or 25 mM caffeine to the culture medium. (**A**) Representative photographs of *Stac3*^+/−^ and *Stac3*^−/−^ myotubes. Scale bars = 200 μm. (**B**) Representative images of myotubes immediately before and after 4-CMC or caffeine treatment. Arrows point to contracting myotubes. (**C**) Percentage of spontaneously contracting myotubes. (**D**) Percentages of 4-CMC and caffeine-induced contracting myotubes. (**E**) Percentages of detached and attached myotubes that contracted in response to 4-CMC or caffeine. Data = mean ± SEM (n =  4 mice per genotype). **P < 0.01.

We compared the contractility of myotubes formed from E18.5 *Stac3*^−/−^ and *Stac3*^+/−^ myoblasts (Fig. 3A). Over 30% of myotubes formed from *Stac3*^+/−^ myoblasts displayed spontaneous contraction, whereas none of those formed from *Stac3*^−/−^ myoblasts was observed to contract (Fig. 3B). In response to 5 mM 4-CMC or 25 mM caffeine, 90% of *Stac3*^+/−^ myotubes contracted, but only 60% of *Stac3*^−/−^ myotubes contracted (*P* = 0.05) (Fig. 3B,D). These data further indicated that defective EC coupling was a major reason but not the only reason that *Stac3*-deleted myotubes could not contract. Interestingly, among the *Stac3*^−/−^ myotubes that contracted in response to 4-CMC or caffeine treatment, approximately 50% of them detached from the plates following the contraction, whereas only 10% of *Stac3*^+/−^ myotubes detached following 4-CMC or caffeine-stimulated contraction (Fig. 3E).

### RyR agonists-induced increases in intracellular calcium in *Stac3*-deleted myotubes

**Figure 4:**
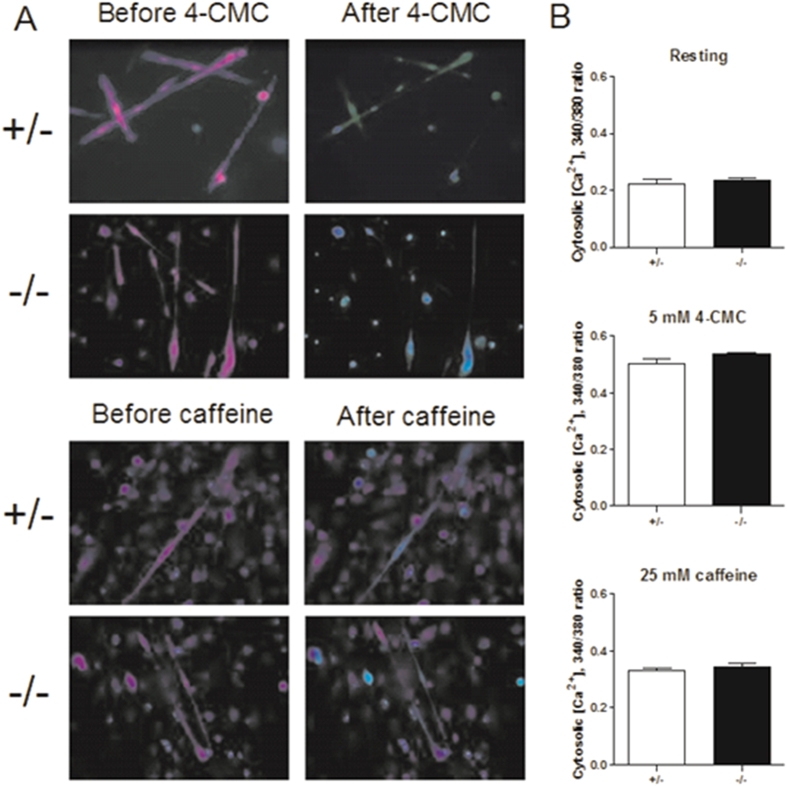
Caffeine- and 4-CMC-induced calcium release in *Stac3*^+/−^ and *Stac3*^−/−^ myotubes. Myotubes were loaded with the fluorescent calcium indicator Fura-2. Fluorescence emission activated by light at 340 nm and 380 nm was recorded by a microscope. (**A**) Representative images of *Stac3*^+/−^ and *Stac3*^−/−^ myotubes loaded with Fura-2 at 3 seconds before and 1 second after 5 mM 4-CMC or 25 mM caffeine treatment. (**B**) Resting and 4-CMC- and caffeine-induced peak intracellular calcium concentrations. Data = mean ± SEM (n ≥ 7 myotubes from 4 mice per genotype).

Reduced contractility in *Stac3*-deleted skeletal muscle could be due to reduced function of RyR or reduced calcium storage in the SR. We determined these possibilities by comparing 4-CMC- and caffeine-induced increases in intracellular calcium in *Stac3*^+/−^ and *Stac3*^−/−^ myotubes (Fig. 4A). As can be seen from Fig. 4B, neither the concentration of intracellular calcium before nor that after stimulation by 5 mM 4-CMC or 25 mM caffeine was different between *Stac3*^+/−^ and *Stac3*^−/−^ myotubes (Fig. 4B). These data indicated that *Stac3* knockout did not affect the function of RyR or the amount of releasable calcium in the SR.

### Fiber composition in *Stac3*-deleted hindlimb muscles

**Figure 5:**
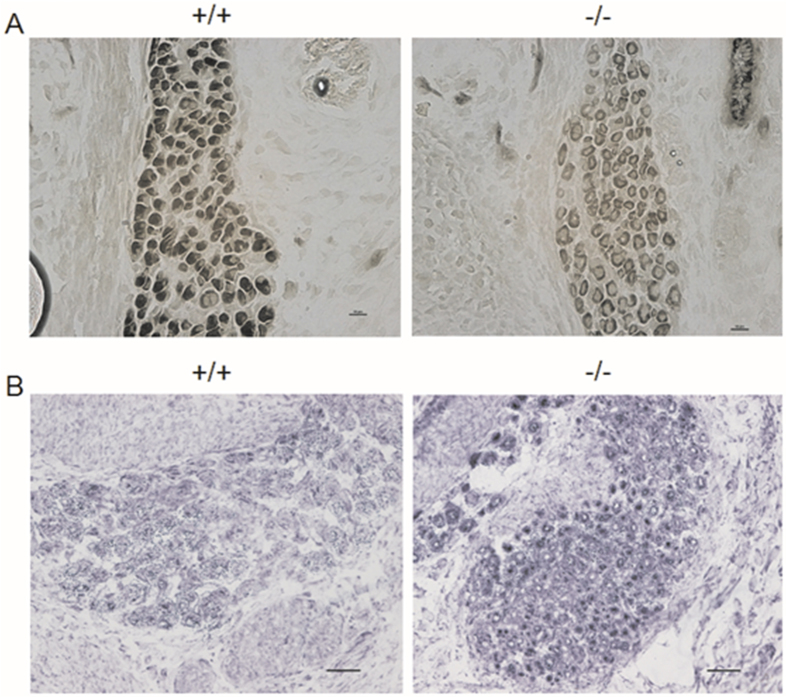
Enzymatic staining of E18.5 *Stac3*^+/+^ and *Stac3*^−/−^ hindlimb muscles. (**A**) Myosin-ATPase staining (pH 10.2). The *Stac3*^−/−^ TA muscle stained lighter than *Stac3*^+/+^ TA muscle. Scale bars = 10 μm. (**B**) NADH-TR staining. In this staining, *Stac3*^−/−^ TA fibers stained darker than *Stac3*^+/+^ TA fibers. Scale bars = 20 μm.

Based on myosin-ATPase and NADH-TR staining, E18.5 *Stac3*^−/−^ hindlimb muscles contained more slow fiber-like myotubes than *Stac3*^+/+^ hindlimb muscles (Fig. 5A,B). Myosin-ATPase staining also revealed that myonuclei, which did not contain myosin and hence stained pale, were localized peripherally in *Stac3*^+/+^ myotubes but centrally in *Stac3*^−/−^ myotubes (Fig. 5A). RT-qPCR analyses revealed that slow fiber markers, such as troponin T type 1 (*Tnnt1*), myosin heavy chain 7 (*Myh7*), myocyte specific enhancer factor 2C (*Mef2c*), peroxisome proliferator-activated receptor gamma coactivator 1 alpha (*Ppargc1a*), and myogenin (*Myog*) were expressed at greater levels in *Stac3*^−/−^ than *Stac3*^+/+^ muscles, whereas fast fiber markers such as *Tnnt3*, *Myh3*, *Myh4*, *Myh8*, and alpha actinin 3 (*Actn3*) were expressed at lower levels in *Stac3*^−/−^ than *Stac3*^+/+^ muscles (Table 1). Overall, these histological and gene expression data indicated that hindlimb muscles from *Stac3*^−/−^ fetuses were more like slow muscles and less like fast muscles than those from *Stac3*^+/+^ fetuses.

## Discussion

Our previous study showed that the mouse *Stac3* gene is specifically expressed in skeletal muscle and that *Stac3* knockout mice died at birth^12^. Our present study showed that hindlimb muscles from *Stac3*-deleted mouse fetuses barely contracted in response to electrostimulation or high [K^+^]-induced membrane depolarization but that they contracted in response to the RyR agonists 4-CMC and caffeine. These results demonstrate that *Stac3*-deleted hindlimb muscles were defective in EC coupling, a finding consistent with that from *Stac3*-deleted diaphragm^11^. However, our study also showed that 4-CMC or caffeine-induced maximal tension in *Stac3*-deleted hindlimb muscles was only 60% of that in normal hindlimb muscles. This result indicates that although defective EC coupling was the major cause, it was not the only cause for the lost contractibility in *Stac3*-deleted hindlimb muscles. In other words, both EC coupling and post EC coupling defects, which could be troponin Ca^2+^ binding defects, less ATP, or defective myofibril protein assembly or expression, contributed to the reduced contractility in *Stac3*-deleted hindlimb muscles.

Discrepant from our study, a post EC coupling defect was not suggested by the response of the *Stac3*-deleted diaphragm to 4-CMC^11^. This discrepancy could be due to different concentrations of 4-CMC used between the studies. In the study by Nelson *et al*., 1 mM 4-CMC elicited similar contraction in *Stac3*-deleted and normal diaphragms^11^. In the present study, we found that while tension induced by 1 mM 4-CMC in *Stac3*-deleted hindlimb muscles was not different from that induced by 5 mM 4-CMC or 25 mM caffeine, tension induced by 1 mM 4-CMC in normal muscles was only 70% of that induced by 5 mM 4-CMC or 50% of that induced by 25 mM caffeine in the same muscles. Our result suggests that 1 mM 4-CMC is insufficient to reveal differences in maximal tension between *Stac3*-deleted and normal muscles and hence insufficient to reveal post EC coupling defects in *Stac3*-deleted muscles. It remains to be determined why it takes less 4-CMC to induce maximal tension in *Stac3*-deleted muscles than in normal muscles. We speculate that either *Stac3*-deleted muscles are more permeable to 4-CMC than normal muscles or that ryanodine receptors are more sensitive to 4-CMC in *Stac3*-deleted muscles than in normal muscles. A membrane difference between *Stac3*-deleted and normal muscles is suggested by the observation that *Stac3*-deleted myotubes were more inclined to detach from the plates than control myotubes upon contraction. The possibility that *Stac3* deletion increased the sensitivity of ryanodine receptors to 4-CMC is, however, not supported by the data that 4-CMC-induced increase in intracellular calcium was not different between *Stac3*-deleted and control myofibers. Two RyR isoforms, RyR1 and RyR3, are expressed in skeletal muscle^8^. RyR3 and RyR1 differ in sensitivity to 4-CMC and caffeine^22^,^23^. The hindlimb muscles express RyR1, but the diaphragm expresses RyR3 in addition to RyR1^8^,^24^. Mouse hindlimb muscles contain predominantly fast fibers instead of the mixture of slow fiber and fast fiber in the diaphragm^19^. These differences in RyR isoform and fiber type could also contribute to the different responses of hindlimb muscles and diaphragm to 4-CMC and caffeine.

Theoretically, reduced RyR expression or function, reduced SR storage of calcium, reduced calcium binding capacity of troponin C, reduced ATP availability, and reduced function of contractile apparatus could each reduce the contractility of skeletal muscle. Our study showed that in response to the RyR agonists 4-CMC and caffeine, similar amounts of calcium were released from the SR in *Stac3*-deleted and control myotubes. This result, consistent with the finding from Stac3-deleted diaphragm^11^, does not support the possibility that *Stac3* deletion reduces muscle contractility by reducing the function of RyR or the amount of releasable calcium stored in the SR. In this study, we found that hindlimb muscles from *Stac3*-deleted mouse fetuses were more like slow muscles than control hindlimb muscles. This result supports the possibility that *Stac3* deletion stimulated slow fiber differentiation or inhibited fast fiber differentiation and thereby reduced muscle contractibility because fast muscles contract with greater force than slow muscles^19^. Reduced muscle activity would stimulate fiber differentiation toward fast muscle^26^,^27^,^28^. Thus, it is unlikely that the effect of *Stac3* deletion on fiber type differentiation was secondary to that on muscle contractility. Skeletal muscle of neonatal *Stac3*-deleted mice contained an abnormally high percentage of centrally localized nuclei and disorganized myofibrils^12^. These abnormalities may be or may be not related to lack of contraction. The SH3 domain is a versatile protein-protein interaction domain^29^,^30^. It is plausible to speculate that while STAC3 mediates EC coupling through interaction with DHPR^13^,^14^, it mediates fiber differentiation, myofibril assembly, and nuclear location through other unknown protein partners.

## Methods

### Animals and genotyping

The generation of heterozygous *Stac3* mutant mice has been described before^12^. These mice were mated to generate homozygous *Stac3* mutant mice. Mice were genotyped as previously described^12^. Homozygous *Stac3* mutant (*Stac3*^−/−^), heterozygous (*Stac3*^+/−^) and wild-type (*Stac3*^+/+^) mouse fetuses at embryonic day 18.5 (E18.5) or E17.5 were used in this study. All protocols involving mice were approved by the Virginia Tech Institutional Animal Care and Use Committee and were carried out in accordance with the approved guidelines.

### Muscle contractility tests

Pregnant mice were euthanized by CO_2_ inhalation followed by cervical dislocation. The fetuses were quickly dissected out of the uterus and euthanized by decapitation on ice. The right hindlimb was skinned and separated from the body at the hip and knee joints in physiological salt solution (PSS: 150 mM NaCl, 4 mM KCl, 2 mM CaCl_2_, 1 mM MgCl_2_, 5 mM HEPES pH 7.4, and 5.6 mM glucose). The hindlimb preparation was then incubated in a chamber of PSS between a clamp and an arm of Dual-Mode Level Servomotor System (300B, Aurora Scientific Inc., Aurora, Ontario, Canada). The PSS was maintained at 30 °C and was constantly bubbled with 95% O_2_ and 5% CO_2_ as previously described^31^. The servomotor arm and stepper motor were controlled by the Dynamic Muscle Control software (DMC Version 4.1.6, Aurora Scientific) to obtain the tension output data.

A pair of *Stac3*^−/−^ and *Stac3*^+/−^ hindlimb muscle preparations were tested at the same time. The treatments were electric stimulation (20 v, 500 μsec; single or frequent), 80 mM KCl, 1 mM and 5 mM 4-CMC, and 25 mM caffeine. The concentrations of KCl, 4-CMC, and caffeine used in this study were based on previous studies^11^,^32^. There was a 1 min interval between two consecutive electric stimulations. There was a 5 min PSS wash out interval between two chemical treatments. Resting tension was set at 0.5 g. At the end of each test, muscles were gently blotted dry with Kimwipes and weighed to the nearest 0.1 mg using an A-200D electronic analytical balance (Denver Instruments, Denver, Colorado). Twitch and tetanic tensions were normalized to muscle mass.

### Myoblast isolation and culture

This procedure was performed as previously described^33^,^34^. Hindlimb muscles from E18.5 mouse fetuses were minced with razors and digested in Ham’s F-10 medium (2 ml/g tissue) supplemented with 1.5 U/ml collagenase B (Roche Diagnostics Corp., Indianapolis, Indiana), 2.4 U/ml dispase (Roche), and 2.5 mM CaCl_2_ at 37 °C for 2 × 10 min. The tissue slurry was gently pipetted to release cells between the two digestions. At the end of the second 10-min digestion, the tissue slurry was mixed with 10 volumes of Ham’s F-10 medium supplemented with 0.1% FBS (Atlanta biologicals, Norcross, Georgia) and passed through a 40 μm nylon filter. The filtrate was centrifuged at 350 × *g* for 5 min. The pelleted cells were resuspended in myoblast culture medium consisting of Ham’s F-10 medium, 20% FBS, 5 ng/ml bFGF (Thermo Fisher Scientific Inc., Waltham, Massachusetts), and antibiotic-antimycotic (ABAM) (Thermo Fisher Scientific), and plated on a non-coated dish for 20 min to allow the fibroblasts to attach. The supernatant in the dish, which contained unattached primary myoblasts, was then transferred to a collagen type I (Sigma-Aldrich, St. Louis, Missouri) coated dish and cultured in a 37 °C incubator with 5% CO_2_ for 24 h. Subsequently, the culture medium was transferred to a 15 ml centrifuge tube while 1 ml of PBS was added to the same dish. The dish was hit firmly against a table top to dislodge the cells. The dish was treated this way for 5–10 min to allow all myoblasts to disassociate from the dish. All the liquid from the dish was collected to the original 15 ml tube. The collection was centrifuged at 350 × *g* for 5 min. The cell pellet was resuspended with myoblast culture medium and plated to a new collagen-coated dish. This preplating procedure was repeated 3 times to enrich myoblasts.

### Myotube contractility analysis

Myoblasts prepared above were plated at 60% confluence on collagen coated 24-well plates and induced to differentiate in medium composed of DMEM, 2% horse serum, and ABAM. Videos of myotubes were taken using a CKX41 inverted microscope connected to a QColor3 OLYMPUS digital camera (Olympus Corp., Shinjuku, Tokyo, Japan). To observe spontaneous contraction in myotubes, 1-minute videos were taken of *Stac3*^+/−^ and *Stac3*^−/−^ myotubes in differentiation medium. To determine the effect of 4-CMC and caffeine on myotube contraction, 5 mM 4-CMC or 25 mM caffeine was added to the medium immediately before 1-minute videos were taken. Contracting or non-contracting myotubes were counted by analyzing the videos. Myotubes that detached from the plates at one end or both ends as a result of contraction were considered as detached myotubes, and those s that remained attached to the plate at both ends following contraction were considered as attached myotubes.

### Calcium imaging

This procedure was performed as previously described^35^,^36^. Briefly, myotubes formed above were loaded with 5 μM fura-2 AM (Thermo Fisher Scientific), a fluorescent calcium indicator, for 40 min in the dark at 37 °C. Myotubes were then rinsed with pre-warmed DMEM twice, each for 10 min. Myotubes were incubated in calcium-free ringer buffer (100 mM NaCl, 6 mM KCl, 2 mM NaHCO_3_, pH 7.4) for 30 min at room temperature to allow complete de-esterification of intracellular AM esters. Fluorescence was measured using an Eclipse Ti fluorescent microscope system with the pre-set fura-2 channel (Nikon Corp., Chiyoda, Tokyo, Japan). At least 3 myotubes were imaged at the same time. To induce the release of calcium from the SR, myotubes were treated with 5 mM 4-CMC or 25 mM caffeine, and the fluorescence was recorded from 1 min before to 5 min after the treatment was added. Intracellular calcium concentration was calculated as the ratio of fura-2 fluorescence excited by ultraviolet light at 340 nm (calcium bound) to that excited at 380 nm (calcium unbound). The 340 nm to 380 nm ratio before the treatment was used as the resting ratio.

### Myosin-ATPase and nicotinamide adenine dinucleotide-tetrazolium reductase (NADH-TR) staining

Left hindlimbs from E18.5 mouse fetuses were immersed in optimal cutting temperature compound (OCT) on a cork sheet and were immediately frozen in liquid nitrogen pre-cooled isopentane. Bones were kept to help locate limb muscles. The muscle samples were left in a Leica CM 1800 cryostat at −20 °C for 20 min for thermal adaptation. Cross-sections of 8 μm thick were cut and mounted on Fisher Superfrost Plus slides (Thermo Fisher Scientific). Myosin-ATPase staining was performed as described^37^. Briefly, muscle sections were pre-incubated for 15 min in sodium barbital buffer containing 20 mM sodium barbital and 36 mM CaCl_2_ at pH 10.2 and then incubated for 45 min in sodium barbital buffer containing 3.5 mM ATP, 20 mM sodium barbital, and 18 mM CaCl_2_ at pH 9.4. Muscle sections were rinsed in 1% CaCl_2_ for 3 × 1 min, and then immersed in 2% CoCl_2_ for 2 min. The sections were then stained with 1% (NH_4_)_2_S for 20 sec followed by 5 rinses with ddH_2_O. Stained sections were dehydrated in 50%, 70%, 80%, 90% and 100% ethanol, cleared in xylene, and then mounted in 50% xylene and 50% Canada balsam mixture (Thermo Fisher Scientific).

The NADH-TR staining was performed as previously described^37^. Briefly, muscle sections were incubated at 37 °C for 10 min and then with 1 mg/ml nitroblue tetrazolium (Sigma-Aldrich) and 0.4 mg/ml β-NADH (Sigma-Aldrich) in 50 mM Tris-HCl buffer, pH 7.3, at 37 °C for 30 min. After 5 × 5 min washes in a graded acetone series (30%, 60%, 90%, 60% and 30%), muscle sections were washed with ddH_2_O and mounted in prolong gold mounting medium (Life Technologies Corp., Carlsbad, California) and cover-slipped for imaging with an Eclipse TS100 microscope (Nikon Corp.).

### Total RNA isolation and RT-qPCR

Hindlimb muscles from E17.5 mouse fetuses were homogenized in TRI reagent (Molecular Research Center, Inc., Cincinnati, Ohio) using a TissueLyser II (Qiagen, Hilden, Germany). The remaining steps followed the manufacturer’s instruction (Molecular Research Center). Concentration and purity of extracted RNA were determined using a NanoDrop 2000 Spectrophotometer (Thermo Fisher Scientific). For first-strand cDNA synthesis, 0.5 μg of total RNA and 0.5 μg random primer (Promega Corp., Madison, Wisconsin) were adjusted to 5 μl with DEPC-treated water and heated to 70 °C for 5 min to denature the RNA. The RNA-primer mixture was then added with 4 μl of 5× reverse transcription reaction buffer, 4.8 μl of 25 mM MgCl_2_, 3.7 μl of DEPC-treated water, 1 μl of 10 mM dNTP, 0.5 μl of RNase inhibitor and 1 μl of ImProm-II reverse transcriptase (Promega). The reaction was incubated at 25 °C for 5 min, 42 °C for 60 min, and then at 70 °C for 15 min. Quantitative PCR (qPCR) was performed on 1 μl of cDNA product in a total volume of 10 μl containing 5 μl of SyberGreen PCR Master Mix (Life Technologies Corp.) and 0.2 μM gene-specific forward and reverse primers (Table 2) under 36 cycles of 15 sec at 95 °C and 1 min at 60 °C on a CFX96 Touch Real-Time PCR Detection System (Bio-Rad Laboratories, Inc., Hercules, California). Primers were designed using the Primer3 software (MIT, Cambridge, Massachusetts). The specificity of all primers was verified by 2% agarose gel electrophoresis and DNA sequencing of PCR products. The efficiency of all primers was determined by analyzing the standard curve of serially diluted cDNA. The amplification efficiency of the primers used in this study was from 90% to 110%. The relative abundance of a mRNA to 18S rRNA was calculated using the ΔΔCt method^38^. The expression levels of all mRNAs were presented as relative values to the average expression level of *Mb* mRNA in Stac3^−/−^ muscles, which was normalized to 1.

### Statistics

Student’s *t* test was used to determine the statistical significance of the differences between two groups. ANOVA followed by Tukey’s test was used to examine the statistical significance of differences between more than two groups. Before ANOVA or Student’s *t* test, homogeneity of variance between groups was analyzed using the F test. If unequal variances were detected, data were log-transformed. A difference was considered statistically significant when *P* < 0.05. All data were presented as mean ± standard error of the mean (SEM).

## Additional Information

**How to cite this article**: Cong, X. *et al*. Defective excitation-contraction coupling is partially responsible for impaired contractility in hindlimb muscles of *Stac3* knockout mice. *Sci. Rep*. **6**, 26194; doi: 10.1038/srep26194 (2016).

## Figures and Tables

**Table 1:** Relative expression levels of select mRNAs in E17.5 Stac3^+/+^ and Stac3^−/−^ hindlimb muscles.

Gene	Fiber type	Stac3^*+/+*^	Stac3^−/−^	*P*-value
*Actn3*	fast	1082.3 ± 167.8^1^	104.6 ± 23.5	0.01
*Myh1*	fast	521.4 ± 29.8	720.4 ± 183.0	0.36
*Myh2*	fast	149.3 ± 27.7	597.7 ± 62.6	0.00
*Myh3*	fast	119343.7 ± 14203.8	98082.6 ± 31645.7	0.56
*Myh4*	fast	165.2 ± 22.3	56.9 ± 24.5	0.02
*Myh8*	fast	220750.7 ± 21934.3	109088.1 ± 30579.6	0.03
*Tnnt3*	fast	24999.9 ± 5187.0	12890.6 ± 4005.5	0.12
*Mb*	slow	1.1 ± 0.25	1.0 ± 0.21	0.71
*Mef2c*	slow	2571.2 ± 345.9	3868.5 ± 911.1	0.23
*Myog*	slow	1324.4 ± 35.7	2913.5 ± 403.5	0.09
*Myh7*	slow	8061.4 ± 946.8	13432.9 ± 5375.0	0.39
*Ppargc1a*	slow	323.0 ± 53.3	414.8 ± 79.0	0.37
*Tnnt1*	slow	8050.3 ± 646.1	13513.4 ± 3592.5	0.23

^1^Data = mean ± SEM (n = 4 mice). *Actn3*, alpha actinin 3; *Myh*, myosin heavy chain; *Tnnt3*, troponin T type 3; *Mb*, myoglobin; *Mef2c*, myocyte specific enhancer factor 2C; *Myog*, myogenin; *Ppargc1a*, peroxisome proliferator-activated receptor gamma coactivator 1 alpha; *Tnnt1*, troponin T type 1.

**Table 2:** Nucleotide sequences of primers used in this study.

Gene	Direction	Primer sequence	GenBank Accession #
*Actn3*	Forward	5′-ATATCGTGAACACCCCCAAA-3′	NM_013456
	Reverse	5′-TCCACTCCAACAGCTCACTG-3′	
*Mb*	Forward	5′-ATGTGAGGGCCAGAGAAAGG-3′	NM_001164047
	Reverse	5′-TCCAGGTACTTGACCGGGAT-3′	
*Mef2c*	Forward	5′-AGAAGTGCAGAGGGAACGAA-3′	NM_001170537
	Reverse	5′-CGCTCATCCATTATCCTCGT-3′	
*Myog*	Forward	5′-CGGCTGCCTAAAGTGGAGAT-3′	NM_031189
	Reverse	5′-AGGCCTGTAGGCGCTCAA-3′	
*Myh1*	Forward	5′-AGTCCCAGGTCAACAAGCTG-3′	NM_030679
	Reverse	5′-CACATTTGCTCATCTTTGG-3′	
*Myh2*	Forward	5′-AGTCCCAGGTCAACAAGCTG-3′	NM_001039545
	Reverse	5′-GCATGACCAAAGGTTTCACA-3′	
*Myh3*	Forward	5′-CGCAGAATCGCAAGTCAATA-3′	NM_001099635
	Reverse	5′-ATATCTTCTGCCCTGCACCA-3′	
*Myh4*	Forward	5′-AGTCCCAGGTCAACAAGCTG-3′	NM_010855
	Reverse	5′-TTTCTCCTGTCACCTCTCAACA-3′	
*Myh7*	Forward	5′-AGTCCCAGGTCAACAAGCTG-3′	NM_080728
	Reverse	5′-TTCCACCTAAAGGGCTGTTC-3′	
*Myh8*	Forward	5′-AGTCCCAGGTCAACAAGCTG-3′	NM_177369
	Reverse	5′-CCTCCTGTGCTTTCCTTCAG-3′	
*Ppargc1a*	Forward	5′-AATGCAGCGGTCTTAGCACT-3′	NM_008904
	Reverse	5′-TTTCTGTGGGTTTGGTGTGA-3′	
*Rn18s*	Forward	5′-TTAAGAGGGACGGCCGGGGG-3′	NM_003278
	Reverse	5′-CTCTGGTCCGTCTTGCGCCG-3′	
*Tnnt1*	Forward	5′-AAACCCAGCCGTCCTGTG-3′	NM_001277903
	Reverse	5′-TCATCTCCCGACCAGTCTGT-3′	
*Tnnt3*	Forward	5′-GCCCAAGAGGAAGAAGTCCA-3′	NR_001163664
	Reverse	5′-TAGCTGCTGTAGTTGGCACC-3′	

*Actn3*, alpha actinin 3; *Mb*, myoglobin; *Mef2c*, myocyte specific enhancer factor 2C; *Myog*, myogenin; *Myh*, myosin heavy chain; *Ppargc1a*, peroxisome proliferator-activated receptor gamma coactivator 1 alpha; *Rn18s*, 18S ribosomal RNA; *Tnnt1*, troponin T type 1; *Tnnt3*, troponin T type 3.

## References

[b1] Murray L. M. . Selective vulnerability of motor neurons and dissociation of pre- and post-synaptic pathology at the neuromuscular junction in mouse models of spinal muscular atrophy. Hum. Mol. Genet. 17, 949–962 (2008). 18065780 10.1093/hmg/ddm367

[b2] Wu H. T., Xiong W. C. & Mei L. To build a synapse: signaling pathways in neuromuscular junction assembly. Development 137, 1017–1033 (2010). 20215342 10.1242/dev.038711 PMC2835321

[b3] Al-Qusairi L. . T-tubule disorganization and defective excitation-contraction coupling in muscle fibers lacking myotubularin lipid phosphatase. Proc. Natl. Acad. Sci. USA 106, 18763–18768 (2009). 19846786 10.1073/pnas.0900705106 PMC2773964

[b4] Al-Qusairi L. & Laporte J. T-tubule biogenesis and triad formation in skeletal muscle and implication in human diseases. Skelet. Muscle 1(26), 1–26 (2011). 21797990 10.1186/2044-5040-1-26 PMC3156648

[b5] O’Brien R. O. . Exclusion of defects in the skeletal muscle specific regions of the DHPR alpha 1 subunit as frequent causes of malignant hyperthermia. J. Med. Genet. 32, 913–914 (1995). 8592342 10.1136/jmg.32.11.913 PMC1051750

[b6] Pietri-Rouxel F. . DHPR alpha1S subunit controls skeletal muscle mass and morphogenesis. EMBO J. 29, 643–654 (2010). 20033060 10.1038/emboj.2009.366 PMC2830706

[b7] Sutko J. L. & Airey J. A. Ryanodine receptor Ca^2+^ release channels: does diversity in form equal diversity in function? Physiol. Rev. 76, 1027–1071 (1996). 8874493 10.1152/physrev.1996.76.4.1027

[b8] Capes E. M., Loaiza R. & Valdivia H. H. Ryanodine receptors. Skelet. Muscle 1, 18 (2011). 21798098 10.1186/2044-5040-1-18 PMC3156641

[b9] Rebbeck R. T. . Skeletal muscle excitation-contraction coupling: who are the dancing partners? Int. J. Biochem. Cell Biol. 48, 28–38 (2014). 24374102 10.1016/j.biocel.2013.12.001

[b10] Legha W. . stac1 and stac2 genes define discrete and distinct subsets of dorsal root ganglia neurons. Gene Expr. Patterns 10, 368–375 (2010). 20736085 10.1016/j.gep.2010.08.003

[b11] Nelson B. R. . Skeletal muscle-specific T-tubule protein STAC3 mediates voltage-induced Ca^2+^ release and contractility. Proc. Natl. Acad. Sci. USA 110, 11881–11886 (2013). 23818578 10.1073/pnas.1310571110 PMC3718085

[b12] Reinholt B. M., Ge X., Cong X., Gerrard D. E. & Jiang H. Stac3 is a novel regulator of skeletal muscle development in mice. PLoS One 8, e62760 (2013). 23626854 10.1371/journal.pone.0062760 PMC3633831

[b13] Horstick E. J. . Stac3 is a component of the excitation-contraction coupling machinery and mutated in Native American myopathy. Nat. Commun. 4, 1952 (2013). 23736855 10.1038/ncomms2952 PMC4056023

[b14] Polster A., Perni S., Bichraoui H. & Beam K. G. Stac adaptor proteins regulate trafficking and function of muscle and neuronal L-type Ca^2+^ channels. Proc. Natl. Acad. Sci. USA 112, 602–606 (2015). 25548159 10.1073/pnas.1423113112 PMC4299259

[b15] Zaharieva I. T. . Whole exome sequencing in patients with congenital myopathies. Neuromuscul. Disord. 24, 895–895 (2014).

[b16] Bower N. I. . Stac3 Is Required for Myotube Formation and Myogenic Differentiation in Vertebrate Skeletal Muscle. J. Biol. Chem. 287, 43936–43949 (2012). 23076145 10.1074/jbc.M112.361311 PMC3527977

[b17] Ge X., Zhang Y., Park S., Cong X. & Gerrard D. E. Stac3 Inhibits Myoblast Differentiation into Myotubes. Plos One 9, e95926 (2014). 24788338 10.1371/journal.pone.0095926 PMC4005754

[b18] Zhang Y., Cong X., Wang A. & Jiang H. Identification of the STAC3 gene as a skeletal muscle-specifically expressed gene and a novel regulator of satellite cell differentiation in cattle. J. Anim. Sci. 92, 3284–3290 (2014). 24948655 10.2527/jas.2014-7656

[b19] Schiaffino S. & Reggiani C. Fiber types in mammalian skeletal muscles. Physiol. Rev. 91, 1447–1531 (2011). 22013216 10.1152/physrev.00031.2010

[b20] Rousseau E., Ladine J., Liu Q. Y. & Meissner G. Activation of the Ca^2+^ release channel of skeletal muscle sarcoplasmic reticulum by caffeine and related compounds. Arch. Biochem. Biophys. 267, 75–86 (1988). 2848455 10.1016/0003-9861(88)90010-0

[b21] Allen D. G. & Westerblad H. The effects of caffeine on intracellular calcium, force and the rate of relaxation of mouse skeletal muscle. J. Physiol. 487 **(Pt 2)**, 331–342 (1995). 8558467 10.1113/jphysiol.1995.sp020883 PMC1156576

[b22] Rossi R., Bottinelli R., Sorrentino V. & Reggiani C. Response to caffeine and ryanodine receptor isoforms in mouse skeletal muscles. Am. J. Physiol. Cell Physiol. 281, C585–594 (2001). 11443058 10.1152/ajpcell.2001.281.2.C585

[b23] Fessenden J. D. . Divergent functional properties of ryanodine receptor types 1 and 3 expressed in a myogenic cell line. Biophys. J. 79, 2509–2525 (2000). 11053126 10.1016/S0006-3495(00)76492-7 PMC1301134

[b24] Lanner J. T., Georgiou D. K., Joshi A. D. & Hamilton S. L. Ryanodine receptors: structure, expression, molecular details, and function in calcium release. Cold Spring Harb. Perspect. Biol. 2, a003996 (2010). 20961976 10.1101/cshperspect.a003996 PMC2964179

[b25] Schiaffino S. . Three myosin heavy chain isoforms in type 2 skeletal muscle fibres. J. Myscle Res. Cell Motil. 10, 197–205 (1989). 10.1007/BF01739810 2547831

[b26] Caiozzo V. J., Haddad F., Baker M. J. & Baldwin K. M. Influence of mechanical loading on myosin heavy-chain protein and mRNA isoform expression. J. Appl. Physiol. 80, 1503–1512 (1996). 8727533 10.1152/jappl.1996.80.5.1503

[b27] Ohira Y. . Myonuclear domain and myosin phenotype in human soleus after bed rest with or without loading. J. Appl. Physiol. 87, 1776–1785 (1999). 10562622 10.1152/jappl.1999.87.5.1776

[b28] Trappe S. . Human single muscle fibre function with 84 day bed-rest and resistance exercise. J. Physiol. 557, 501–513 (2004). 15064323 10.1113/jphysiol.2004.062166 PMC1665105

[b29] Li S. S. Specificity and versatility of SH3 and other proline-recognition domains: structural basis and implications for cellular signal transduction. Biochem. J. 390, 641–653 (2005). 16134966 10.1042/BJ20050411 PMC1199657

[b30] Saksela K. & Permi P. SH3 domain ligand binding: What’s the consensus and where’s the specificity? FEBS Lett. 586, 2609–2614 (2012). 22710157 10.1016/j.febslet.2012.04.042

[b31] Grange R. W., Gainer T. G., Marschner K. M., Talmadge R. J. & Stull J. T. Fast-twitch skeletal muscles of dystrophic mouse pups are resistant to injury from acute mechanical stress. Am. J. Physiol. Cell Physiol. 283, C1090–1101 (2002). 12225973 10.1152/ajpcell.00450.2001

[b32] Takeshima H. . Excitation-contraction uncoupling and muscular degeneration in mice lacking functional skeletal muscle ryanodine-receptor gene. Nature 369, 556–559 (1994). 7515481 10.1038/369556a0

[b33] Rando T. A. & Blau H. M. Primary mouse myoblast purification, characterization, and transplantation for cell-mediated gene therapy. J. Cell Biol. 125, 1275–1287 (1994). 8207057 10.1083/jcb.125.6.1275 PMC2290930

[b34] Pisaniello A. . The block of ryanodine receptors selectively inhibits fetal myoblast differentiation. J. Cell Biol. 116, 1589–1597 (2003). 10.1242/jcs.00358 12640042

[b35] Bakker A. J., Head S. I., Williams D. A. & Stephenson D. G. Ca^2+^ levels in myotubes grown from the skeletal muscle of dystrophic (mdx) and normal mice. J. Physiol. 460, 1–13 (1993). 8487190 10.1113/jphysiol.1993.sp019455 PMC1175197

[b36] Park S., Scheffler T. L. & Gerrard D. E. Chronic high cytosolic calcium decreases AICAR-induced AMPK activity via calcium/calmodulin activated protein kinase II signaling cascade. Cell calcium 50, 73–83 (2011). 21641034 10.1016/j.ceca.2011.05.009

[b37] Suga T. . Muscle fiber type-predominant promoter activity in lentiviral-mediated transgenic mouse. PLoS One 6, e16908 (2011). 21445245 10.1371/journal.pone.0016908 PMC3060803

[b38] Livak K. J. & Schmittgen T. D. Analysis of relative gene expression data using real-time quantitative PCR and the 2(T)(-Delta Delta C) method. Methods 25, 402–408 (2001). 11846609 10.1006/meth.2001.1262

